# Prognostic biomarkers and molecular pathways mediating *Helicobacter pylori*–induced gastric cancer: a network-biology approach

**DOI:** 10.5808/gi.22072

**Published:** 2023-03-31

**Authors:** Farideh Kamarehei, Massoud Saidijam, Amir Taherkhani

**Affiliations:** 1 Department of Microbiology, Faculty of Medicine, Hamadan University of Medical Sciences, Hamadan 6517838678, Iran; 2 Research Center for Molecular Medicine, Hamadan University of Medical Sciences, Hamadan 6517838678, Iran

**Keywords:** biomarkers, *Helicobacter pylori*, prognosis, protein interaction maps, stomach neoplasms, survival analysis

## Abstract

Cancer of the stomach is the second most frequent cancer-related death worldwide. The survival rate of patients with gastric cancer (GC) remains fragile. There is a requirement to discover biomarkers for prognosis approaches. *Helicobacter pylori* in the stomach is closely associated with the progression of GC. We identified the genes associated with poor/favorable prognosis in *H. pylori*–induced GC. Multivariate statistical analysis was applied on the Gene Expression Omnibus (GEO) dataset GSE54397 to identify differentially expressed miRNAs (DEMs) in gastric tissues with *H. pylori*–induced cancer compared with the *H. pylori*–positive with non-cancerous tissue. A protein interaction map (PIM) was built and subjected to DEMs targets. The enriched pathways and biological processes within the PIM were identified based on substantial clusters. Thereafter, the most critical genes in the PIM were illustrated, and their prognostic impact in GC was investigated. Considering p-value less than 0.01 and |Log2 fold change| as >1, five microRNAs demonstrated significant changes among the two groups. Gene functional analysis revealed that the ubiquitination system, neddylation pathway, and ciliary process are primarily involved in *H. pylori*–induced GC. Survival analysis illustrated that the overexpression of DOCK4, GNAS, CTGF, TGF-b1, ESR1, SELE, TIMP3, SMARCE1, and TXNIP was associated with poor prognosis, while increased MRPS5 expression was related to a favorable prognosis in GC patients. DOCK4, GNAS, CTGF, TGF-b1, ESR1, SELE, TIMP3, SMARCE1, TXNIP, and MRPS5 may be considered prognostic biomarkers for *H. pylori*–induced GC. However, experimental validation is necessary in the future.

## Introduction

Cancer of the stomach is the fifth-frequent carcinoma [1] and the second leading cause of malignancy related deaths worldwide [2-5], with approximately one million new cases each year, which contributes to being a major global health problem. Previous studies have found that gastric cancer (GC) is a heterogeneous disease in which the genetic and epigenetic alterations of vital human genes associated with the cell cycle and DNA repair procedures and environmental factors mediate the occurrence and progression of the disease [6-9].

It has been demonstrated that bacterial pathogens in the human stomach are involved in GC development. The primary human gastric pathogen, *Helicobacter pylori*, has infected more than 50% of the human population. Approximately 5%–15% of *H. pylori*–positive patients reveal gastric disorders ranging from gastritis and metaplasia to gastric carcinoma [10]. *H. pylori* is the leading risk factor for developing GC [11-14] and has been detected in most patients with stomach cancer [11]. The infection of the gastric mucosa caused by *H. pylori* may result in constant inflammation in gastric tissue by promoting the expression of different cytokines (e.g., interleukin 1 beta, interleukin-1 receptor antagonist, and tumor necrosis factor-α), which can lead to enhanced levels of reactive oxygen species, DNA damage, and hyper-activation of tumorigenesis signaling pathways associated with cancer [12-20].

Despite the recent progress of new diagnostic and therapeutic approaches in GC, the mean survival times for advanced stages is not favorable. Due to early diagnosis, the survival rate is approximately 5%–20% in Western countries and 50% in Japan [3,21-24]. In addition, the exact molecular etiology of the disease has not been fully illustrated. By identifying the tumor suppressor genes that are usually down-expressed due to deletion or mutation, as well as discovering the tumor promoter genes associated with gastric carcinoma, the underlying mechanisms of the disease could further be elucidated, and more knowledge would be provided in the diagnostic, prognostic, and therapeutic procedures of GC [23-33].

Cancer biomarkers are differentially expressed molecules in patients with cancer compared to healthy individuals. Some biomarkers are the main reason for abnormal cellular and molecular changes leading to malignancy, and others are secreted in response to the disease. The prognostic markers are used to predict the situation of patients in the future, independent of the treatment obtained and may be used for predicting personalized medicine. In addition, the overall survival rate of patients and cancer recurrence could be expected by identifying prognostic biomarkers [34-36]. During the last decades, several of these markers have been introduced by cancer researchers [37-41]. Therefore, physicians are encouraged to use validated biomarkers for personalized medicine as adjuvant treatment [34].

The small non-coding RNAs contributing to the gene regulatory process at the post-transcriptional stage are named microRNAs (miRNAs). They bind to their specific complementary nucleotides at different regions of the genes [42-48]. Previous studies have demonstrated that miRNAs could either promote or diminish the expression of genes [49,50]. In this regard, miRNAs could enhance their target genes' expression if they bind to the promoter region. However, these small molecules could result in gene silencing if they attach to other parts of the genes, such as 3′ untranslated region (UTR), 5′ UTR, and the coding sequence [50,51]. MiRNAs contribute to gene regulation and play a decisive role in several biological procedures, such as cellular proliferation and differentiation, apoptosis, development, inflammation, carcinogenesis, and metastasis. The abnormal expression of miRNAs in tissues may result in tumorigenesis or vice versa [52]. Therefore, miRNAs have become encouraging molecules in biomarker discovery in cancer research [53-55]. The most significant miRNAs associated with the initiation, progression, and prognosis of GC could be determined by analyzing their expression in gastric normal and tumor tissues [56].

Microarray is high-throughput technology suitable for simultaneously analyzing thousands of gene expression patterns [57]. A large number of variables with a small sample size are characteristics of high-throughput data. Therefore, robust statistical approaches are necessary for analyzing data obtained from microarray, which may result in identifying reliable biomarker candidates. Orthogonal-partial least squares–discriminant analysis (OPLS-DA) is a multivariate statistical method widely used for analyzing high-throughput data, leading to identifying differential variables significantly expressed among classified groups [58].

Reproducibility, also known as repeatability or precision, is the degree to which repeated measurements of an equal amount will display similar or comparable results. Standard deviation, variance, and Pearson correlation coefficient are commonly used to report the reproducibility of a dataset in the microarray technique. For ideally precise technologies, the variance of a measurement is zero [59]. For oligonucleotide arrays such as Agilent, Affymetrix, and Codelink, the Pearson correlation coefficient is calculated as > 0.9 [60,61]. Due to the high-throughput property of the microarray technique, which makes it possible to screen the complete profile of molecules, it has been widely used for miRNA analysis [21,62]. The miRNA expression profiles have demonstrated more stability, accuracy, and reproducibility than mRNA signatures. Because of the high stability of miRNAs in body fluids, they are assigned valuable biomarkers for clinical diagnosis and prognosis of human diseases [63-65]. However, a robust RNA isolation approach is necessary for achieving reliable results. Trizol/TRI-reagent-based isolation has demonstrated reproducible results, leading to considerable miRNA resistance to degradation when properly prepared and stored [66].

In the present study, we exposed differentially expressed miRNAs (DEMs) between *H. pylori*–induced gastric cancerous tissue and non-tumor tissue collected from *H. pylori*–positive patients. Subsequently, the targets of DEMs were determined, and a protein interaction map (PIM) was built and analyzed. The most critical genes in the PIM were identified, and their prognostic impact in GC patients was studied using the GEPIA database. Moreover, the most significant pathways and Gene Ontology (GO) terms deregulated in the *H. pylori*–induced GC were discussed. We followed the methods of Bayat et al. (2021) [67]. Of note, different p-value thresholds were used in this study for various analyses. Notably, Yue et al. [68] additionally used different p-value thresholds in their previous research to identify DEGs in metastasis nasopharyngeal carcinoma (NPC) samples compared to the nonmetastatic specimens (p < 0.01), as well as enriched pathways in NPC (p < 0.05).

## Methods

### Microarray expression data acquisition and analysis

The raw microarray expression dataset of GSE54397 [69] was obtained as a TXT format from the Gene Expression Omnibus (GEO) source [70]. GSE54397 contained 32 observations containing eight *H. pylori*–induced gastric cancerous tissues, eight non-tumor tissues collected from *H. pylori*–positive patients, eight gastric cancerous tissues obtained from *H. pylori*–negative patients, and eight non-tumor tissues collected from *H. pylori*–negative patients. The dataset was based on the GPL15159 platform (Agilent-031181 Unrestricted_Human_miRNA_V16.0_Microarray 030840). To discover novel risk factors in patients affected by *H. pylori*, a new dataset was selected from the GSE54397, which consisted of eight *H. pylori*–induced gastric cancerous tissue samples and eight tissue samples with no cancer signs were achieved from *H. pylori*–positive individuals. This might help to detect GC in infected individuals. Normalization was performed prior to statistical analysis. The OPLS-DA identified the DEMs between two groups using the R version 4.0.2 programming language [71]. The cutoff conditions were set to an absolute Log2 fold change |Log2 FC| > 1 and the p-value less than 0.01 [68,72]. The volcano plot of miRNAs in the two studied groups was achieved using the Shiny apps web-based tool [73]. Moreover, the hierarchical clustering of differential miRNAs was conducted utilizing the R language.

### PIM construction, module detection, and functional analysis

The validated targets of considerable DEMs were determined utilizing the MiRWalk 2.0 [74]. The GO annotation analyses for these targets, including cellular components (CCs) and molecular functions (MFs), were carried out utilizing the ClueGO version 2.5.7 tool [75]. The STRING online database [76] version 11.0 was used to illustrate the interactions between target genes. The single proteins were excluded from the primary PIM before further analysis. The PIM was analyzed using the Cytoscape software [77], leading to the identification of hub genes with the highest degree and betweenness centralities [78]. Moreover, clustering analysis was performed using the MCODE tool. Modules with the following benchmarks were assigned as significant condensed regions: score ≥ 3, depth ≤ 100, k-score = 2, node score cutoff = 0.2, degree ≥ 2, and the minimum number of nodes = 10 [79]. Thereafter, significant pathways and biological processes (BPs) enriched by these modules were studied. The Reactome database [79] and the ClueGO tool were used for pathway and GO annotation analyses, respectively. The minimum number of enriched genes as two, besides the false discovery rate (FDR) as < 0.05 [67,68,80-83], were assigned meaningful for the affected pathways and BP terms in *H. pylori*–induced GC.

### Survival analysis

The Kaplan-Meier curve was generated for the hub genes using the (GEPIA) web server [84] to investigate the prognostic impact of hub markers in gastric carcinoma. Furthermore, the Cox proportional hazards regression model was utilized to determine the corrected hazard ratios (HR) and 95% confidence intervals of hub genes and evaluate the prognostic factors' independence. The prognostic impact of markers with the HR and log-rank test p < 0.05 [67,80-83] were statistically considered meaningful.

### Identifying common DEMs between *H. pylori*–induced gastric cancerous tissues and *H. pylori*–negative specimens

Besides the main dataset which was analyzed in this study (including *H. pylori*–induced gastric cancerous tissues [n = 8] and non-tumor tissues collected from *H. pylori*–positive patients [n = 8]), two other datasets were extracted from the GSE54397 as follows: one of them included *H. pylori*–induced GC samples (n = 8) and *H. pylori*–negative cancerous tissues (n = 8) and the other dataset contained *H. pylori*–positive GC specimens (n = 8) and *H. pylori*–negative normal tissues (n = 8). All three datasets were analyzed using the OPLS-DA algorithm to detect the common DEMs in three different datasets. The DEMs with the criteria of the p-value less than 0.01 and |Log2 FC| more than one were statistically assigned significantly.

### Gene expression evaluation of prognostic markers

The gene expression patterns of prognostic markers in GC were evaluated at the mRNA and protein levels using the GEPIA2 [84] and the Human Protein Atlas (HPA) databases, respectively. The GEPIA2 server provides boxplot analysis using stomach adenocarcinoma tissues (n = 408) and normal gastric specimens (n = 211). The HPA has been developed since 2003 to map all the human proteins in cells, tissues, and organs using various technologies, including antibody-based imaging and mass spectrometry-based proteomics. The HPA, freely available at https://www.proteinatlas.org/ [85], allows researchers to access the expression patterns of the human proteome.

### Ethical approval

The present study was approved by the Ethics Committee of Hamadan University of Medical Sciences, Hamadan, Iran (ethics no. IR.UMSHA.REC.1399.583). No human/animal was used in this study.

## Results

### DEMs in *H. pylori*–induced gastric carcinoma

**Fig. 1.**
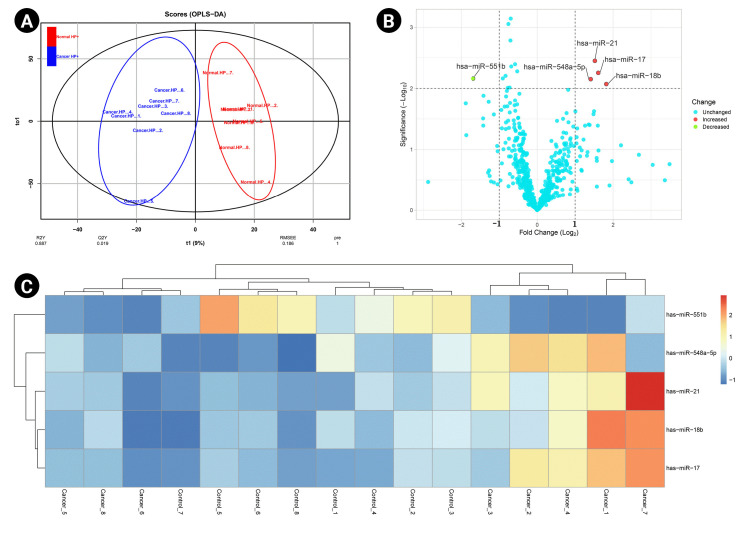
. (A) The score plot in the predictive (x-axis) and orthogonal (y-axis) components of microarray data achieved from the tissue samples using the orthogonal projections to latent structures discriminant analysis. (B) The volcano plot of the miRNAs in *Helicobacter pylori*–induced gastric cancer compared to the non-tumor tissue collected from H. pylori–positive patients. (C) The heat map and hierarchical clustering of differentially expressed miRNAs in the two studied tissues. OPLS-DA, orthogonal-partial least squares–discriminant analysis.

A predictive OPLS-DA model was constructed for the dataset containing *H. pylori*–induced gastric cancerous tissue samples (n = 8) and non-tumor gastric tissue samples from *H. pylori*–positive patients (n = 8). The R2X, R2Y, and Q2 of the OPLS-DA were calculated as 0.344, 0.887, and 0.019, respectively (Fig. 1A). Four overexpressed, and one underexpressed miRNA were indicated to be statistically differential in *H. pylori*–induced GC patients compared to the healthy controls (p < 0.01; |Log2 FC| > 1) (Table 1). Fig. 1B demonstrates the volcano plot of miRNAs in the studied groups. Moreover, Fig. 1C illustrates the heat map of differential miRNAs among case-control samples.

### Protein interaction map, clustering, and functional analyses

**Fig. 2.**
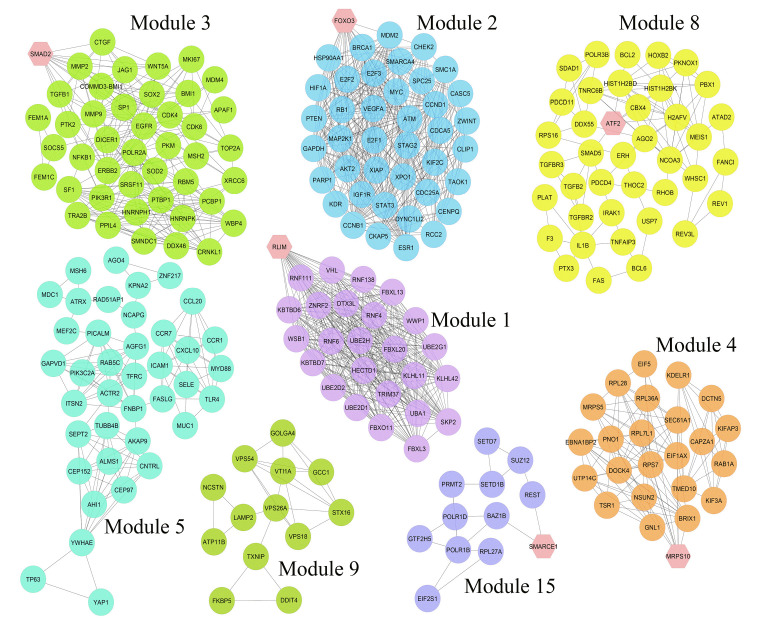
Module analysis. These genes are validated differentially expressed miRNAs-targets in *Helicobacter pylori*–induced gastric cancer tissues than *H. pylori*–positive samples with no cancer symptoms. The interactions between proteins were identified using the STRING knowledge database. The MCODE tool discovered eight substantial clusters in the graph. The hexagons illustrate seed nodes.

**Fig. 3.**
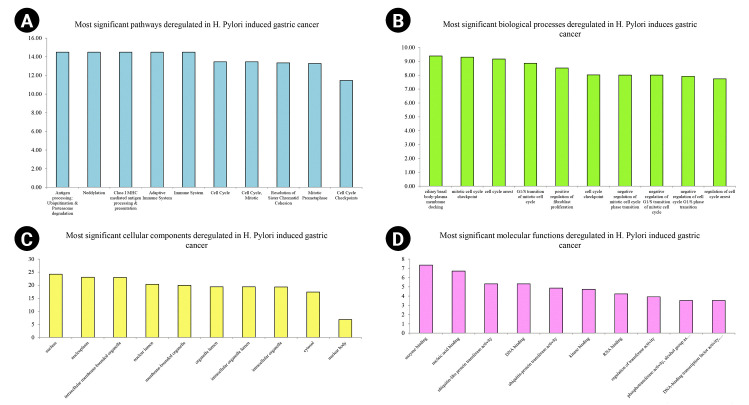
Top-10 significant pathways (A), biological processes (B), cellular components (C), and molecular functions (D) enriched in *Helicobacter pylori*–induced gastric cancer patients regarding their false discovery rate. The x-axis demonstrates the pathway and gene ontology term's names, while the y-axis shows –Log10 of false discovery rate.

**Fig. 4.**
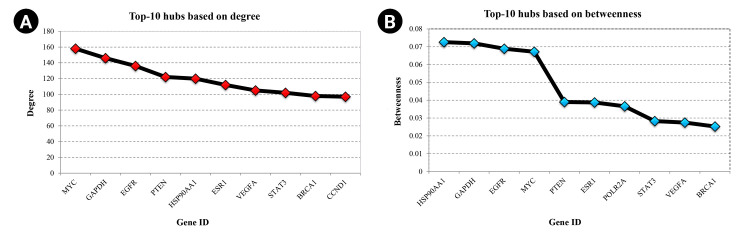
(A) Top-10 hubs based on the degree value. (B) Top-10 hubs according to their betweenness centrality.

Nine hundred seventy genes were determined as experimentally validated targets of DEMs. Therefore, a PIM was constructed based on these genes utilizing the STRING source with a confidence score of ≥0.4. After excluding single nodes, a PIM with 931 proteins and 6,861 interactions was imported into the Cytoscape for further analyses, including functional and structural studies. Eight substantial modules were detected inside the PIM (Fig. 2). Table 2 presents the topological features of each cluster. At an FDR of 0.05, 399 pathways and 224 BPs were significantly enriched in patients with *H. pylori*–induced GC than those with *H. pylori*–positive patients with non-tumor gastric tissue. Moreover, 31 CCs and 51 MFs were affected considerably in *H. pylori*–induced gastric carcinoma (FDR < 0.05) [67,80-83]. The most significant pathways and GO terms enriched in *H. pylori*–induced GC are demonstrated in Fig. 3. In addition to the network analysis results, the average degree and betweenness values of the nodes in the network were 59.85 and 0.0149, respectively. Furthermore, 175 proteins had degree and betweenness centrality values more remarkable than the mean of the network vertexes and therefore, assigned as the most critical genes associated with the etiology of *H. pylori*–induced GC, named hubs (Supplementary Table 1). Fig. 4A and 4B demonstrate the top 10 hub genes regarding their degree and betweenness centralities, respectively.

### Prognostic impact of the hub genes

**Fig. 5.**
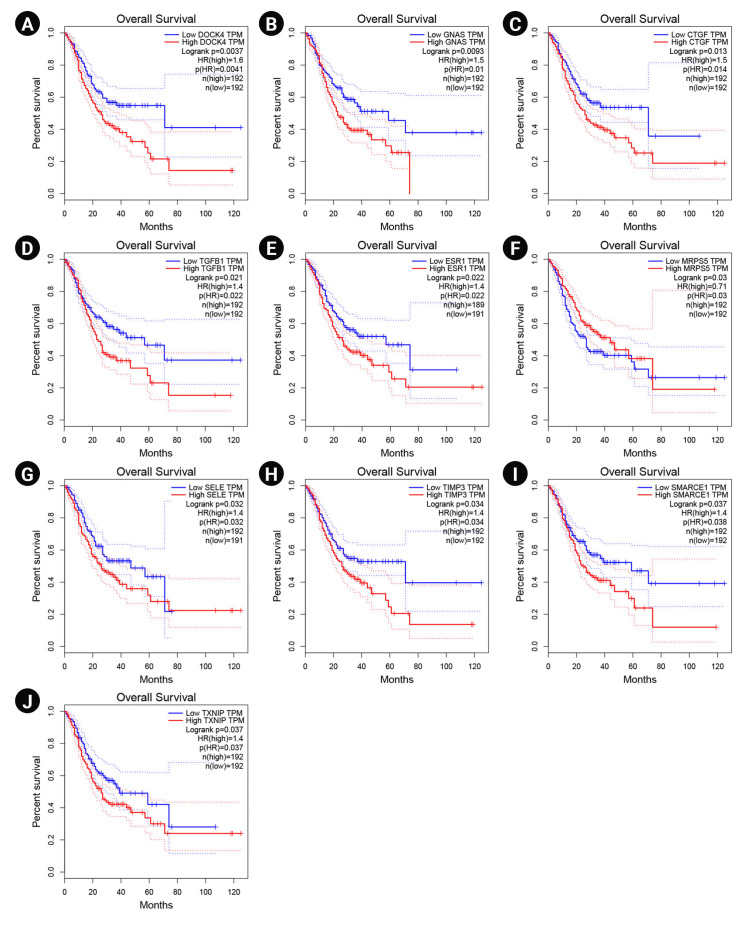
Survival analysis of DOCK4 (A), GNAS (B), CTGF (C), TGF-b1 (D), ESR1 (E), MRPS5 (F), SELE (G), TIMP3 (H), SMARCE1 (I), and TXNIP (J) genes. Blue and red lines demonstrate under and overexpressed markers, respectively. The y-axis and x-axis illustrate the probability of survival and survival months of patients with gastric cancer, respectively. The dotted lines show a 95% confidence interval. TPM, transcripts per million; HR, hazard ration.

The overexpression of DOCK4, GNAS, CTGF, TGF-b1, ESR1, SELE, TIMP3, SMARCE1, and TXNIP significantly revealed a poor prognosis in GC patients. Therefore, these markers may participate in the metastasis and recurrence of GC and could be considered potential cancer markers associated with a dismal prognosis in *H. pylori*–induced gastric carcinoma. In addition, enhanced expression of MRPS5 exhibited a favorable prognosis in GC patients. The Kaplan-Meier curves for these potential prognostic biomarkers are presented in Fig. 5.

### Common DEMs between *H. pylori*–positive GC samples and *H. pylori*–negative specimens

**Fig. 6.**
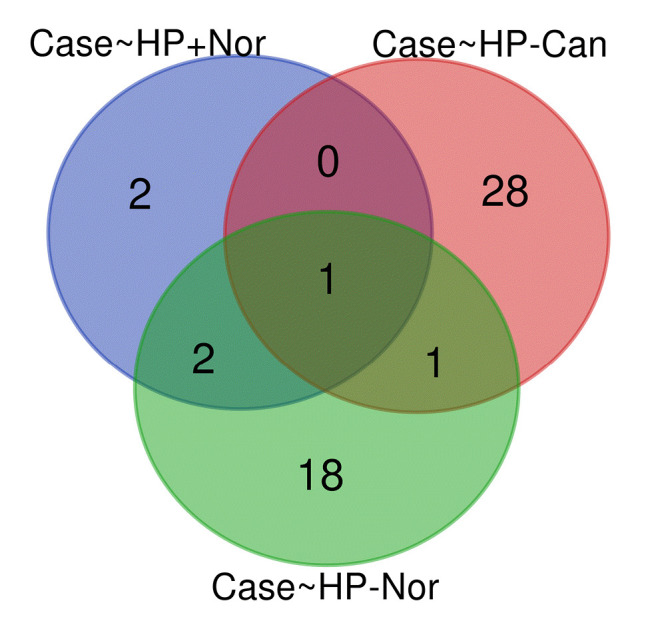
Common differentially expressed miRNAs among *Helicobacter pylori*–induced gastric cancer tissues (case group) and *H. pylori*–negative (HP–) samples. HP+, *H. pylori*–positive; Nor, normal; Can, cancer.

By analyzing three different datasets, 30 DEMs were found in *H. pylori*–positive GC samples compared to *H. pylori*–negative specimens. Also, 22 DEMs were identified in *H. pylori*–induced GC compared with the *H. pylori*–negative healthy controls. Moreover, has-miR-551b was a common DEM in *H. pylori*–induced GC compared to the other *H. pylori*–negative tissues (p < 0.01 and |Log2 FC| > 1) (Table 3). The common DEMs between three different datasets were discovered using the Venn diagrams (Fig. 6).

### Markers expression study

**Fig. 7.**
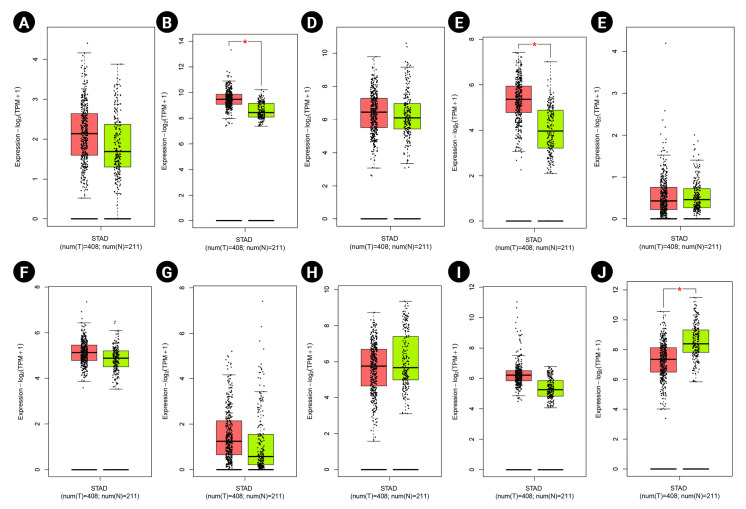
Gene expression patterns at the mRNA level for prognostic markers in gastric cancer (GC) including DOCK4 (A), GNAS (B), CTGF (C), TGFB1 (D), ESR1 (E), MRPS5 (F), SELE (G), TIMP3 (H), SMARCE1 (I), and TXNIP (J). Box plots are based on 408 GC tissues (red color) and 211 healthy gastric samples (green color). TPM, transcripts per million.

**Fig. 8.**
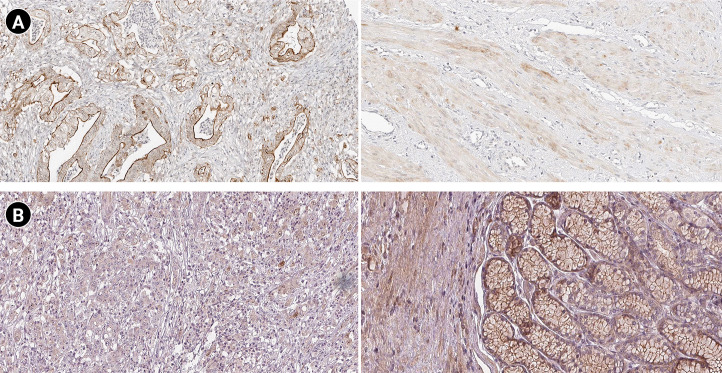
Protein expression patterns of GNAS (A) and TXNIP (B) in gastric cancer. The left and right images demonstrate protein staining in cancerous and healthy tissues, respectively.

According to the boxplot analysis, the mRNA levels of DOCK4, GNAS, TGFB1, SELE, and SMARCE1 demonstrated a considerably higher expression in gastric adenocarcinoma than in healthy controls. CTGF and MRPS5 showed a mild overexpression in GC compared with normal gastric tissues. Besides, TXNIP illustrated a significant underexpression in GC compared to the healthy control specimens (Fig. 7). Based on the histopathological analysis, GNAS exhibited a higher expression in GC specimens than in healthy control tissues (Fig. 8A). As well, TXNIP expression was lower in stomach cancer compared with the normal gastric samples, consistent with boxplot analysis (Fig. 8B).

### Ethical approval

The present study was approved by the Ethics Committee of Isfahan University of Medical Sciences, Isfahan- Iran (ethics no. IR.MUI.RESEARCH.REC.1400.539).

## Discussion

GC is one of the prominent carcinoma-related deaths globally, with a dismal mean survival time, although some progress has been made in the diagnostic and therapeutic approaches. *H. pylori* is the primary human pathogen in the gastric mucosa of almost half of the global population, which participates in developing GC through the regulation of miRNA expression. miRNAs have been noticed as prognostic biomarkers in GC due to their gene regulatory role in cells, such as tumor suppressors and promoter functions [86].

**Fig. 9.**
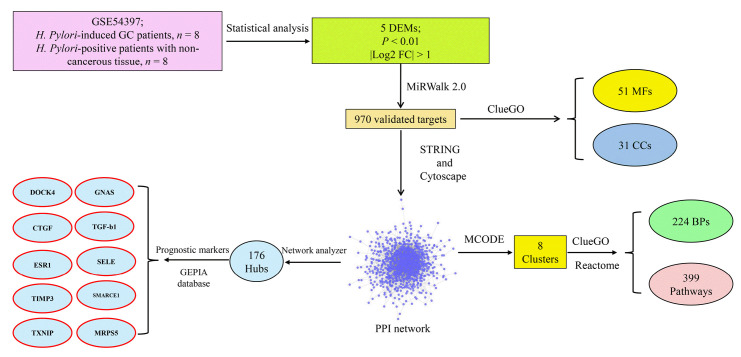
A schematic of the present study's research design and main findings. BP, biological process; CC, cellular component; DEM, differentially expressed miRNA; GC, gastric cancer; MF, molecular function.

The present study revealed that the most substantial modules of the PIM associated with *H. pylori*–induced GC were primarily enriched in the ubiquitination system, neddylation pathway, and ciliary process. Moreover, overexpression of DOCK4, GNAS, CTGF, TGF-b1, ESR1, SELE, TIMP3, SMARCE1, and TXNIP was significantly associated with poor prognosis. At the same time, increased expression of MRPS5 revealed a favorable prognosis in patients with GC. Fig. 9 demonstrates the study design and critical points of the present study.

The ubiquitin-proteasome system is an intracellular protein modification pathway that degrades most proteins in mammalian cells [87]. It is executed through ubiquitin-activating enzymes E1, ubiquitin-conjugating enzyme E2, and ubiquitin ligase E3 [88,89]. According to previous studies, dysregulation of E3 ubiquitin enzymes and impropriety targeting of the proteins by E3 leads to many disorders, such as cancer metastasis, including GC [90-93]. Thus, blocking the ubiquitin-proteasome pathways administers a novel approach to treating carcinomas [94].

The Cullin-Ring ligases (CRLs) are involved in the targeted degradation of approximately 20% of cellular proteins [95,96]. It has been reported that the misregulation of CRLs, especially CRL1, is linked to many human disorders, such as cancer [97,98]. Therefore, CRL1 ligase is a potential drug target for cancer treatment [99-102]. Notably, the neddylation of cullins is required to form active CRLs E3 ligases. In the neddylation pathway, the protein NEDD8 is transferred onto the lysine of one of the cullin subunits by the NEDD8-conjugating enzyme and NEDD8-activating enzyme (NAE) [22,27,103,104]. According to previous studies, the neddylation pathway is upregulated in many human malignancies. Therefore, targeting the neddylation pathway by inhibiting NAE has been demonstrated as an effective anticancer strategy in preclinical and clinical settings [98,101,105-108].

A cilium or cilia (plural) are immotile hair-like structures assembled from the cell membrane of almost all eukaryotic cells. Several studies have linked tumorigenesis, tumor-relevant defects, and the deregulation of mammalian target of rapamycin signaling proteins localized at cilia [109,110]. Although the initiation of cancer depends on the presence of cilia in medulloblastoma [111], the loss of cilia has been reported in different types of malignancies such as renal cell carcinoma [112], breast cancer [113,114], and basal cell carcinoma [115].

The dedicator of cytokinesis protein 4 (DOCK4) regulates cell-cell adhesion junction and plays a role in cell metastasis [116-120]. In addition, this gene contributes to many biological processes in mammalians, including tumor cell malignant transformation, proliferation, and metastasis [121]. Overexpression of DOCK4 has been linked to tumor progression and poor survival rate in patients with breast cancer [122] and liver cancer patients [123].

According to a previous study, *GNAS* mutation could result in tumorigenesis by activating the Wnt signaling pathway [124]. Gastric adenocarcinoma of the fundic gland type (GAFG) is a subclass of gastric adenocarcinoma [125]. Most GAFGs occur in non-atrophic gastric mucosa without *H. pylori* infections are infrequent branching and anastomosing tubules lined with basophilic columnar cells with mild nuclear atypia resembling chief cells [126]. In addition, pyloric gland adenoma (PGA) is another subtype of GC characterized by atrophic mucosa with constant inflammation as the cause of *H. pylori* infection [126,127]. Previous studies have linked the GNAS and KRAS (GTPase KRas protein) mutations and the development of PGA [128]. Survival analysis demonstrated that GNAS overexpression is significantly associated with a poor prognosis in GC patients. Besides, the boxplot and immunohistochemical analyses confirmed the GNAS overexpression in GC tissues at the mRNA and protein levels.

It has been shown that higher expression of CTGF in gastric carcinoma contributes to peritoneal and local lymph node metastasis [129,130]. Moreover, CTGF suppression inhibits cellular proliferation and metastasis in GC [131]. Li et al. [132] reported that a higher mRNA expression of CTGF was positively associated with local invasion in GC cells. In addition, lower mRNA levels of CYR61 and CTGF revealed a more prolonged survival time in GC patients. Patients with enhanced CTGF, CYR61 and NOV mRNA levels demonstrated dismal mean survival times.

Previous studies have linked the polymorphism of TGF-b1 C-509T and the risk of promoting GC [133-136]. Chang et al. [69] demonstrated that the TGF-b1-509T allele contributed to TGF-b1 enhanced expression. Its overexpression in normal tissue revealed a potential promoting effect related to *H. pylori* infection, leading to the progression of intestinal-type GC. Moreover, TGF-b1 was overexpressed in the antrum of *H. pylori*–positive patients [137], and the TGF-b1 expression was significantly reduced after treating *H. pylori* infection [138]. Jayapal and Melendez [139] reported that the increased expression of several cytokines, such as TGF-b1, in the gastric antrum is associated with the infection caused by *H. pylori*. The feedback loop, including TGF-b1, Smad-7, and CTGF, could be involved in the pathogenesis of *H. pylori*–associated gastritis. CTGF is a downstream effector of TGF-b [140], so overexpression of TGF-b1 and CTGF can cause acute and maintained fibrosis [141].

Trefoil factor 1 (TFF1) is involved in gastric tumor suppression [142-144]; it is lost in more than 50% of GC cells because of epigenetic silencing, TFF1 deletions, or its transcription factors downregulation [145-147]. In breast cancer, estrogen receptor 1 (ESR1) regulates the TFF1 expression. According to the results of other studies and our study, it may be speculated that the enhanced ESR1 expression in GC patients with a dismal outcome is due to the reaction of enhanced tumor size. However, this requires validation.

Zhou et al. [148] reported lower protein and mRNA expression levels of MRPS5 in cancerous gastric tissue compared with the adjacent tissues. This was executed by utilizing the Human Protein Atlas immunohistochemistry source [149] and quantitative real-time polymerase chain reaction (qRT-PCR) analysis. According to previous studies and the results achieved from survival analysis, it may be hypothesized that MRPS5 acts as a tumor suppressor gene in GC and may be assigned as a favorable prognostic gene in GC patients. However, more experiments are required to verify the above.

E-selectin, the protein encoded by the *SELE* gene, mediates the progression and invasion of GC through different mechanisms, including promoting angiogenesis by activating the Src-PI3K pathway [150,151]. A positive correlation has been observed in GC between the serum expression levels of circulating E-selectin and tumor progression and metastasis, leading to a poor prognosis [152-155]. Liarmakopoulos et al. [150] demonstrated that the E-selectin S128R C allele was related to dismal survival in GC patients.

The aberrant expression of matrix metalloproteinase-3 (MMP-3) and tissue inhibitor of metalloproteinase-3 (TIMP-3) is potentially associated with metastasis in several carcinomas such as NPC [156], cervical cancer [157], breast cancer [158], lung cancer [159], and colon cancer [160]. The Kaplan-Meier analysis from the GEPIA database showed that the overall survival rate of the GC patients with overexpression of TIMP3 was lower than GC patients with down expression of TIMP3. This may be due to the response of increased cancer cell invasion and metastasis, although this requires confirmation.

Liu et al. [161] reported that SMARCE1 was overexpressed in GC cell lines and tissues. In addition, the upregulation of SMARCE1 was significantly linked with the malignant clinicopathological features of GC patients. Moreover, Liu et al. [161] reported that the enhanced SMARCE1 expression was considerably related to a dismal prognosis in GC patients (p < 0.01). As well the enhanced SMARCE1 expression significantly induced the GC cell invasion *in vitro*, as well as tumorigenesis *in vivo*.

TXN gene promotes hypoxia-inducible factor-1α, leading to vascular endothelial growth, tumor angiogenesis, and drug resistance [162]. The enhanced TXN expression in tumors has been linked to a worse survival rate of patients in several carcinomas [163,164]. The TXN-interacting protein (TXNIP) suppresses the connection between TXN and other factors. Therefore, TXNIP upregulation attenuates the activity of TXN, leading to decreased proliferation and cell cycle progression in tumor cells [165,166]. Kwon et al. [167] demonstrated that the loss of TXNIP in a mouse model promoted *H. pylori*–induced GC. Evidence suggests that different ethnicities might affect the gene expression profile in patients with GC [168]. Based on the boxplot and histopathological analyses, it was revealed that TXNIP is downregulated in GC patients at mRNA and protein levels.

Our study had certain limitations. Only eight *H. pylori*–induced gastric cancerous tissue samples and eight non-tumor tissue samples from patients infected with *H. pylori* were involved within the GSE54397; therefore, our sample size was not large. Including the more significant number of observations in the dataset may elevate the statistical potential and illustrate more considerable DEMs related to the etiology of *H. pylori*–induced GC. Besides, the miRNAs profiled in the present study may not support all miRNAs. In future experiments, large targeted groups are needed to verify these markers.

It is suggested that five miRNAs are differentially expressed in patients with *H. pylori*–induced GC compared to *H. pylori*–positive patients with non-cancerous tissue (p-value less than 0.01 and |Log2 FC| > 1). In addition, PIM analysis revealed 176 hubs as proteins considerably taking part in the etiology of *H. pylori*–induced GC. Survival analysis showed that the overexpression of DOCK4, GNAS, CTGF, TGF-b1, ESR1, SELE, TIMP3, SMARCE1, and TXNIP, could lead to a dismal overall survival rate. At the same time, the upregulation of MRPS5 was associated with a good prognosis in GC patients. Therefore, these genes may be cancer markers for prognosis in *H. pylori*–induced GC. However, more investigations are required in the future to examine the tissue expression of these genes in *H. pylori*–induced GC and to understand better the exact role that these molecules serve in the carcinogenesis of the disease. In addition to the PIM functional analysis results, we found that the most substantial clusters were primarily enriched in the ubiquitination system, neddylation pathway, and ciliary processes.

## Figures and Tables

**Table 1.** Five of the miRNAs were assigned as differential in patients with *Helicobacter pylori*–induced gastric cancer compared to H. pylori–positive patients with non-cancerous tissue, identified by microarray analysis

miRNA ID	FC disease/control	ABS Log2 FC	p-value
hsa-miR-21	2.86	1.51	0.00353
hsa-miR-18b	2.27	1.18	0.00846
hsa-miR-548a-5p	2.66	1.41	0.00706
hsa-miR-17	3.06	1.61	0.00556
hsa-miR-551b	0.31	1.71	0.0069

FC, fold change; ABS, absolute.

**Table 2.** Details of eight substantial clusters in the protein interaction map related to *Helicobacter pylori*–induced gastric cancer

Cluster No.	MCODE score	No. of nodes	No. of edges	Seed node	Seed degree	Seed betweenness
1	26	26	325	RLIM	32	0.0051
2	20.35	41	407	FOXO3	63	0.0104
3	10.837	44	233	SMAD2	64	0.0118
4	8.957	24	103	MRPS10	23	0.0042
5	6.474	39	123	NA	NA	NA
8	4.923	40	96	ATF2	33	0.0026
9	4.167	13	25	NA	NA	NA
15	3.091	12	17	SMARCE1	21	0.0029

NA, not available.

**Table 3.** Differentially expressed miRNAs in three datasets selected from GSE54397

Groups of study	Total No. of common DEMs	miRNA ID
Case vs. HP+ Normal & Case vs. HP– Cancer & Case vs. HP– Normal	1	hsa-miR-551b
Case vs. HP+ Normal & Case vs. HP– Normal	2	hsa-miR-21, hsa-miR-17
Case vs. HP– Cancer & Case vs. HP– Normal	1	hsa-miR-934
Case vs. HP+ Normal	2	hsa-miR-548a-5p, hsa-miR-18b
Case vs. HP– Cancer	28	hsa-miR-1321, hsa-miR-765, hsa-miR-3667-5p, hsa-miR-654-5p, kshv-miR-K12-3, ebv-miR-BART16, hsa-miR-4300, hsa-miR-595, hsa-miR-492, hsa-miR-519e, hcmv-miR-UL112, hsa-miR-4296, hsa-miR-3621, hsa-miR-320c, hsa-miR-1182, hsa-miR-630, hsa-miR-939, ebv-miR-BHRF1-1, hsa-miR-28-3p, hsa-miR-3679-5p, ebv-miR-BART12, hsa-miR-1291, hsa-miR-664, hsa-miR-3187-3p, hsa-miR-708, hsa-miR-3663-5p, kshv-miR-K12-7, hsa-miR-1915
Case vs. HP– Normal	18	hsa-miR-25, hsa-miR-508-3p, hsa-miR-548d-5p, hsa-miR-3117-3p, hsa-miR-1305, hsa-miR-335, hsa-miR-645, hsa-miR-3127-5p, hsa-miR-892b, hsa-miR-1288, ebv-miR-BART19-3p, hsa-miR-3125, hcmv-miR-UL22A, hsa-miR-204, hsa-miR-375, hsa-miR-604, hsa-miR-148a, hsa-let-7c_v16.0

miRNA, microRNA; DEM, differentially expressed miRNA; HP+, *Helicobacter pylori*–positive; HP–, *H. pylori*–negative.

## Supplementary Materials

Supplementary Table 1. A total of 176 hub genes were identified in the protein-protein interaction network associated with *Helicobacter pylori*–induced gastric cancer
