# Supplementary material for: Prognostic biomarkers and molecular pathways mediating Helicobacter pylori–induced gastric cancer: a network-biology approach
Source: Genomics Inform. 2023 Mar 31;21(1):e8. doi: 10.5808/gi.22072 (PMC10085735; doi:10.5808/gi.22072)
Supplement: Supplementary Table 1. — A total of 176 hub genes were identified in the protein-protein interaction network associated with Helicobacter pylori–induced gastric cancer [file gi-22072-Supplementary-Table-1.pdf]

Supplementary Table 1. A total of 176 hub genes were identified in the protein-protein interaction network associated with *Helicobacter pylori*–induced gastric cancer

Gene ID	Degree	Betweenness
HSP90AA1	120	0.072591
GAPDH	146	0.071941
EGFR	136	0.068877
MYC	158	0.067190
PTEN	122	0.039014
ESR1	112	0.038769
POLR2A	95	0.036577
STAT3	102	0.028272
VEGFA	105	0.027468
BRCA1	98	0.025286
MDM2	89	0.020723
CCNB1	79	0.018972
SKP2	65	0.017959
XPO1	68	0.017482
PIK3R1	60	0.017095
VHL	54	0.016519
ATM	91	0.015747
SMARCA4	80	0.014885
IL1B	57	0.014162
CCND1	97	0.014090
TFRC	35	0.013930
SP1	62	0.013610
ERBB2	77	0.012662
VCP	34	0.012483
UBE2D1	58	0.011993
DERL1	20	0.011922
PKM	40	0.011899
SMAD2	64	0.011850
SOD2	42	0.011791
DICER1	54	0.011626
PABPC1	48	0.011613
MMP2	53	0.010776
UBA1	46	0.010644
MMP9	67	0.010560
NCOA3	47	0.010507
TLR4	54	0.010436
FOXO3	63	0.010416
RB1	58	0.010395
RAB1A	31	0.010175

POLR3B	30	0.010172
NFKB1	54	0.009862
NIPBL	36	0.009669
HNRNPK	51	0.009300
MUC1	22	0.009211
DDB1	33	0.009074
FBXO11	40	0.009021
PTBP1	48	0.008920
YWHAE	43	0.008749
WNT5A	43	0.008619
YAP1	36	0.008606
ATRX	49	0.008478
MAP2K1	50	0.008319
KDR	56	0.008295
SEC61A1	26	0.008166
H2AFV	63	0.007963
UBE2D2	49	0.007865
RHO	21	0.007849
SETD2	44	0.007789
IGF1R	52	0.007741
PARP1	59	0.007705
CRKL	29	0.007652
SOX2	53	0.007561
RAP1A	27	0.007258
MON2	17	0.007004
ACTR2	32	0.006993
MEF2C	29	0.006958
RHOB	28	0.006792
CHEK2	59	0.006675
WWP1	46	0.006673
ARF6	25	0.006630
GNAQ	27	0.006537
RHOQ	21	0.006440
VPS26A	16	0.006296
AGFG1	24	0.006238
PDCD11	31	0.006200
HNRNPH1	43	0.006080
VIM	24	0.005794
PPIF	15	0.005559
CAPZA1	18	0.005518
CKAP5	35	0.005460
E2F3	37	0.005441
MAPK13	29	0.005382

HIF1A	57	0.005355
PCBP1	36	0.005329
KIF2C	39	0.005298
TGFBR2	37	0.005208
UBE2H	31	0.005191
SUZ12	48	0.005164
RLIM	32	0.005109
SETD1B	27	0.005043
ICAM1	39	0.004994
GNAS	18	0.004993
SYNCRIP	32	0.004881
PTK2	39	0.004875
AKAP9	28	0.004806
VPS54	17	0.004776
RAB6A	23	0.004738
HOXA9	15	0.004663
MAP3K1	25	0.004650
OPTN	21	0.004609
AKT2	44	0.004557
DYNC1LI2	27	0.004466
TGFB1	47	0.004426
USP7	29	0.004347
MTA1	24	0.004320
LATS1	16	0.004297
JAG1	27	0.004270
CSE1L	17	0.004264
E2F1	50	0.004211
MRPS10	23	0.004203
RAB5C	25	0.004188
RPS16	39	0.004161
TPM1	19	0.004122
RNF4	42	0.004010
TRA2B	36	0.003997
FAS	30	0.003895
ACTN4	22	0.003895
FMR1	29	0.003888
LAMP2	16	0.003825
DOCK4	26	0.003820
TOP2A	51	0.003776
TAOK1	20	0.003758
PIK3C2A	19	0.003737
XRCC6	40	0.003658
CLU	21	0.003598

FASLG	29	0.003496
PLD1	20	0.003448
SRPK2	20	0.003424
CEP152	16	0.003423
MSH2	39	0.003419
HIST1H2BD	50	0.003399
HIST1H2BK	50	0.003399
PRKCA	31	0.003380
UBR5	21	0.003369
SPCS3	15	0.003347
SIRT2	24	0.003272
CTGF	38	0.003227
STAG2	34	0.003217
ALMS1	20	0.003194
THOC2	24	0.003144
EBNA1BP2	26	0.003118
PPARA	18	0.003100
TIMP3	22	0.003092
RNF111	32	0.003086
RPL27A	39	0.003044
BBS10	22	0.003040
ZNRF2	30	0.003023
SELE	20	0.003016
SMARCE1	21	0.002948
REV1	15	0.002899
KPNA2	37	0.002893
AGO2	39	0.002887
REV3L	16	0.002838
MRPS5	25	0.002831
TXNIP	17	0.002799
KAT6A	21	0.002760
EIF4A2	31	0.002759
FEM1C	16	0.002752
LARS	15	0.002732
SMAD7	34	0.002723
RSF1	18	0.002710
ATF2	33	0.002671
RPL28	23	0.002664
SMG1	23	0.002621
EIF1AX	28	0.002621
ZNF217	15	0.002614
APC	21	0.002596
BCL6	23	0.002594

PPIL4	15	0.002537
SMC1A	39	0.002506
CUX1	19	0.002503
MDC1	37	0.002469
FBXL3	37	0.002453
KDM5A	23	0.002452
CDK4	55	0.002450
ATAD2B	16	0.002418
